# Eosinophilic ascites due to severe eosinophilic ileitis

**DOI:** 10.4103/1742-6413.70408

**Published:** 2010-09-17

**Authors:** Namrata Setia, Peter Ghobrial, Pantanowitz Liron

**Affiliations:** Department of Pathology Pittsburgh, PA, USA; 1 Department of Radiology, Baystate Medical Center, Tufts University School of Medicine, Springfield, MA; 2 Department of Pathology, University of Pittsburgh Medical Center, Pittsburgh, PA, USA

**Keywords:** Ascites, cytology, eosinophilia, gastroenteritis, ileitis, peritoneal fluid, serosa

## Abstract

**Background:**

There is a broad etiology for effusion eosinophilia that includes allergic, reactive, infectious, immune, neoplastic, and idiopathic causes. We report and describe the cytomorphologic findings of a rare case of eosinophilic ascites due to severe eosinophilic ileitis.

**Case Presentation:**

A 17-year-old male manifested acutely with eosinophilic ascites due to severe biopsy-proven subserosal eosinophilic ileitis. Isolated peritoneal fluid submitted for cytologic evaluation revealed that 65% eosinophils were present in a bloody background. The patient responded to corticosteroids, with complete resolution of his ascites.

**Conclusion:**

Eosinophilic gastroenteritis with subserosal involvement should be added to the list of causes for eosinophils in peritoneal fluid. The finding of eosinophilic ascites, with appropriate clinical and laboratory findings, may warrant the need to perform laparoscopic intestinal biopsies to confirm the diagnosis.

## INTRODUCTION

The occurrence of eosinophils in peritoneal fluid is a rare phenomenon. This may be attributed to various allergic (asthma, hypersensitivity reactions), infectious (typically parasitic), immune (collagen vascular diseases), and neoplastic (leukemia/lymphoma) diseases, as well as hypereosinophilic syndrome, chronic peritoneal dialysis, or idiopathic causes.[1–3] Air and/or blood in body cavities due to trauma, postoperative, or spontaneous (e.g., pneumothorax) causes may also provoke eosinophilia.[4–7] The eosinophilic reaction in such cases is thought to be caused by the introduction of dust particles. Foreign bodies like ventriculoperitoneal shunts or chest tubes are another source of eosinophils in the body fluids.[1] Eosinophilic pleural effusions have been reported to also be caused by drugs, pulmonary embolism, and asbestos exposure.[6] Eosinophilic (pleural) effusions are defined as those that contain at least 10% eosinophils in the fluid white cell differential count.[6,8] While eosinophilic ascites due to eosinophilic gastroenteritis (EGE) has previously been documented,[9–13] the cytologic findings of such ascitic fluid have not been well characterized. In most cases, the diagnosis of EGE is established by histological examination.[14,15] We report a rare case of a young male with eosinophilic ascites due to severe biopsy-proven eosinophilic ileitis. The cytomorphology and clinicopathologic findings in this rare case are described.

## CASE REPORT

A 17-year-old male with a past medical history significant for asthma and gastrointestinal reflux disease presented with a 1-week history of intermittent abdominal pain, nausea, bilious emesis, and bloody diarrhea. Physical examination revealed periumbilical and right lower quadrant tenderness without peritoneal signs. Laboratory tests showed a white blood cell count of 11,500 cells/mm^3^ with 31% eosinophils (the absolute eosinophil count was 3,600/mm^3^). A stool test for ova and parasites was negative. Quantitative immunoglobulins were normal. Serology for Toxocara and human immunodeficiency virus were negative. Strongyloides antibodies were equivocal. erythrocyte sedimentation rate (ESR), antinuclear antibody (ANA), and anti-neutrophil cytoplasmic antibody (ANCA) antibodies were normal. Ultrasound examination performed at the time of admission revealed moderate ascites, dependent in the right lower and upper abdominal quadrant. Computerized tomography (CT) of his abdomen demonstrated thickening of the terminal ileum and ascites. No free air in the abdomen was noted. The ascitic fluid was aspirated under CT guidance and sent for cytological evaluation. A hepatobiliary iminodiacetic acid scan to track the flow of bile was normal. An esophagogastroduodenoscopy (EGD) and colonoscopy with mucosal biopsies were performed, which showed a notable increase in esophageal eosinophils, but no colitis. This was followed by laparoscopic examination to obtain small bowel serosa and mesenteric biopsies. During laparoscopy, petechiae were identified on the serosa of the ileum. Following a diagnosis of eosinophilic ileitis with associated eosinophilic ascites (see below), intravenous steroid treatment was started. The patient responded very well to therapy and was discharged on oral prednisone, which was eventually tapered and stopped. A follow-up ultrasound of the abdomen demonstrated virtually complete resolution of his intraabdominal fluid.

### Cytologic findings

**Figure 1:**
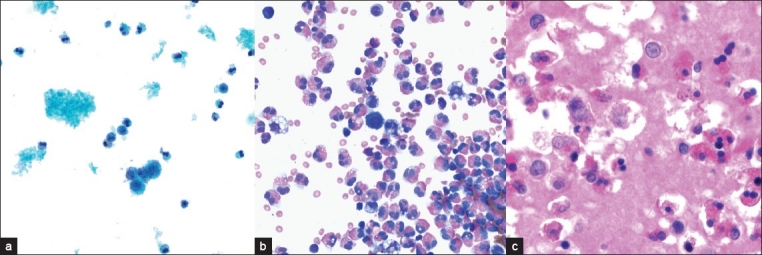
Ascitic fluid showing increased numbers of eosinophils with a) Pap stain (ThinPrep, original magnification ×600) and b) Wright-Giemsa stain (cytospin, original magnification ×600) and c) in a cell block preparation (hematoxylin and eosin stain, original magnification ×600)

Straw-colored ascitic fluid was obtained and submitted to both the cytopathology laboratory in Cytolyt and the hematology laboratory. Fluid analysis [Table 1] was remarkable for 65% eosinophils. ThinPrep slides were stained with a Papanicolaou stain, a cytospin with a Wright-Giemsa stain, and a cell block was prepared and stained with hematoxylin and eosin. The peritoneal fluid revealed an abundance of mature eosinophils [Figure 1] present in a bloody background. Malignant cells or microorganisms were not identified. Microbiology cultures of the ascitic fluid were negative for bacteria, mycobacteria, and fungal organisms.

**Table 1:** Chemical and cellular analysis of ascitic fluid

Appearance and color	Straw, hazy
Total protein	4.5 g/dl
Albumin	3.0 g/dl
Glucose	100 mg/dl
LDH	152 Units/L
Triglycerides	58 mg/dl
Red blood cells	7,360/mm^3^
White blood cells	6,000/mm^3^
Eosinophils	65%
Lymphocytes	6%
Monocytes	25%
Other cell types	4%

### Histopathologic findings

**Figure 2:**
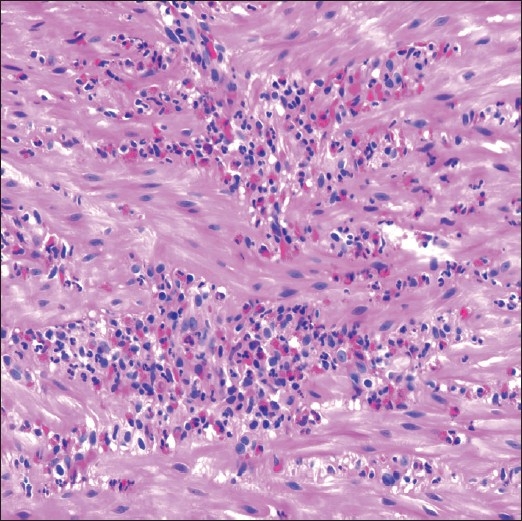
Histologic section from a laparoscopic ileal biopsy demonstrating aggregates of eosinophils within the muscularis propria (hematoxylin and eosin stain, original magnification ×600)

Multiple gastrointestinal biopsies were obtained as described above. Occasional intraepithelial eosinophils were present in the proximal and distal esophageal mucosa. Gastric and colorectal biopsies did not show increased eosinophils. However, within the ileum, there were numerous eosinophils present in the muscularis propria and the serosa [Figure 2], diagnostic of eosinophilic enteritis. A mesenteric lymph node demonstrated reactive lymphoid hyperplasia with numerous sinusoidal eosinophils and associated Charcot-Leyden crystals.

## DISCUSSION

EGE represents a family of diseases including eosinophilic esophagitis, gastritis, enteritis, and colitis. Peripheral blood eosinophilia may be seen in a large proportion of these patients. Eosinophilic lymphadenopathy, as was evident in this patient’s mesentery, has only rarely been reported in association with EGE. In the gastrointestinal tract, the gastric antrum and proximal small bowel are the sites most likely to be involved.[16] Eosinophilic enteritis of the terminal ileum occurs only infrequently. EGE can be classified based on the location of the eosinophilic infiltrate in the gastrointestinal wall and associated symptoms. The histopathologic diagnosis of EGE requires the presence of ≥20–25 eosinophils per high-power field on microscopic examination.[14,15] Eosinophils in EGE frequently infiltrate the gastrointestinal mucosa and, only rarely, the muscular or subserosal layers.[9] Patients with mucosal disease present mainly with functional bowel disease (e.g., abdominal pain, diarrhea, bleeding, malabsorption, protein-losing enteropathy), whereas those with muscular involvement tend to present more with bowel thickening and associated surgical obstruction.[14] Those with subserosal disease may have associated eosinophilic ascites and peripheral eosinophilia. Eosinophilic ileitis in our patient was characterized by numerous eosinophils extending from the muscular wall of the small bowel to the subserosa, even involving the serosal surface of the terminal ileum. Subserosal disease is reported to be the least common form of EGE, and quite distinct from the other subtypes with respect to clinical presentation. In such cases, patients generally manifest with or without symptoms of functional bowel disease, isolated ascites, a higher incidence of peripheral blood eosinophilia, a personal and family history of allergies, and excellent response to steroids.[14] Not surprisingly, superficial mucosal biopsies obtained by EGD and colonoscopy in subserosal EGE are likely to be nondiagnostic. The radiologic findings of EGE are usually nonspecific, although prior reports mention the presence of bowel wall thickening as the most common finding in all subtypes of EGE, and ascites in subserosal EGE.[17] Unlike eosinophilic pleural effusion, which requires >10% eosinophils of the total white blood cells, there are no defined criteria for the diagnosis of eosinophilic ascites. As alluded to the above, there is a broad etiology for a peritoneal effusion eosinophilia. The presence of increased eosinophils in peritoneal fluid should prompt clinicians to exclude obvious causes (e.g., pneumoperitoneum, chronic peritoneal dialysis, congestive heart failure), neoplasia, as well as more rare entities such as vasculitis and a ruptured hydatid cyst.[3,18] In our case, severe eosinophilic ileitis with serosal involvement was the likely cause of this patient’s eosinophilic ascites. Considering the bloody background in our case, however, the possibility of blood contamination was raised as a potential source of eosinophilia. The exact mechanism of ascites is unclear, but likely involves inflammatory mediators (e.g., eosinophil major basic protein, interleukins, and eotaxin).[19] Eosinophils are round to oval cells, ranging in size from 10 to 15 μm, containing segmented nuclei with two to three lobes without nucleoli, and are characterized by notable eosinophilic cytoplasmic granules. However, on a Pap stain, these cytoplasmic granules may be inconspicuous, in which case their lobated nuclei are more of a reliable cytomorphological feature. Charcot-Leyden crystals, considered to be a morphologic hallmark of eosinophil-related disease,[20] have seldom been reported in serous fluids.[21] Such crystals are best demonstrated when either the eosinophilic fluid or a wet film has stood for at least 24 h.[22] As mast cells and basophils often accompany eosinophils, a few of these cells may be seen in cases of body fluid eosinophilia.[3]

In summary, we report an unusual case of eosinophilic ascites due to severe subserosal eosinophilic ileitis associated with allergy, eosinophilic lymphadenopathy, and peripheral blood eosinophilia. EGE with subserosal involvement should therefore be added to the list of causes for eosinophils in peritoneal fluid. The finding of eosinophilic ascites with enteritis, in association with appropriate clinical and laboratory findings, should prompt the need to perform laparoscopic intestinal biopsies of suspicious areas in place of EGD and colonoscopic biopsies.[23]

## COMPETING INTEREST STATEMENT BY ALL AUTHORS

No competing interest to declare by any of the authors.

## AUTHORSHIP STATEMENT BY ALL AUTHORS

Each author acknowledges that this final version was read and approved. All authors qualify for authorship as defined by ICMJE http://www.icmje.org/#author Each author participated sufficiently in the work and takes public responsibility for appropriate portions of the content of this article.

## ETHICS STATEMENT BY ALL AUTHORS

Our institution does not require approval from the Institutional Review Board for a case report without identifiers.

## EDITORIAL / PEER-REVIEW STATEMENT

To ensure integrity and highest quality of CytoJournal publications, the review process of this manuscript was conducted under a double blind model(authors are blinded for reviewers and reviewers are blinded for authors)through automatic online system.
